# Synthesis and Bioconjugation of Gold Nanoparticles as Potential Molecular Probes for Light-Based Imaging Techniques

**DOI:** 10.1155/2007/29817

**Published:** 2007-08-29

**Authors:** Raja Gopal Rayavarapu, Wilma Petersen, Constantin Ungureanu, Janine N. Post, Ton G. van Leeuwen, Srirang Manohar

**Affiliations:** ^1^Biophysical Engineering Group, Institute for Biomedical Technology (BMTI), Faculty of Science and Technology, University of Twente, P.O. Box 217, 7500 AE Enschede, The Netherlands; ^2^Molecular Cell Biology Group, Polymer Chemistry and Biomaterials, Institute for Biomedical Technology (BMTI), Faculty of Science and Technology, University of Twente, P.O. Box 217, 7500 AE Enschede, The Netherlands

## Abstract

We have synthesized and characterized gold nanoparticles (spheres and rods) with optical extinction bands within the “optical imaging window.” The intense plasmon resonant driven absorption and scattering peaks of these nanoparticles make them suitable as contrast agents for optical imaging techniques. Further, we have conjugated these gold nanoparticles to a mouse monoclonal antibody specific to HER2 overexpressing SKBR3 breast carcinoma cells. The bioconjugation protocol uses noncovalent modes of binding based on a combination of electrostatic and hydrophobic interactions of the antibody and the gold surface. We discuss various aspects of the synthesis and bioconjugation protocols and the characterization results of the functionalized nanoparticles. Some proposed applications of these potential molecular probes in the field of biomedical imaging are also discussed.

## 1. INTRODUCTION

Optical imaging encompasses a multitude of techniques for the elucidation of morphology, molecular function, and metabolism of tissue with the general objective of detecting, diagnosing, staging, and treatment monitoring of disease. Progression of disease is usually accompanied by changes in physiology and pathology that are manifested as location-specific changes in optical properties thereby providing contrast for optical imaging to study disease.

Optical imaging techniques span the range from surface to bulk imaging systems with applications ranging from “optical biopsies” to full human breast imaging with resolutions that cover the microscopic to macroscopic. Some important imaging techniques for superficial tissue imaging are confocal microscopy [1], two-photon microscopy [2], and optical coherence tomography (OCT) [3]. Techniques that permit subsurface to deep imaging are diffuse optical imaging (DOT) [4] and photoacoustic imaging [5].

The interaction of visible and near-infrared (NIR) light with tissue is dominated by

- absorption processes which are due to the presence of various chromophores such as hemoglobin, oxy-hemoglobin, melanin, water, and lipids [6];
- scattering processes due to the cell membrane and cell structures such as the nucleus, mitochondria, lysosomes [6].

Penetration of light in tissue is dependent on the extent of the two processes above and is low in the high-energy visible region of the spectrum. This is due to high absorption by hemoglobin and severe light scattering. In the wavelength regime between 600 nm and 1100 nm, absorption and scattering losses are minimal permitting high-light penetration. This is the so-called “optical imaging window” which is exploited for deep imaging in tissue [7].

The sensitivity and specificity of optical imaging techniques to visualize a pathological disorder are governed by contrast: the ability of the disease to differentially scatter or absorb light compared with nonpathological tissue and background noise. This native or endogenous contrast may not be sufficient and in any case, the interactions of light with tissue are not disease-specific. Therefore, there is a role for exogenously administered contrast enhancing agents which have affinity for the disease site through biochemical interactions, providing not only sensitive but also disease-specific signals.

Contrast agents for optical imaging thus far have near-infrared dyes based on cyanine dyes [8] such as Indocyanine Green [9], but in the last few years, gold nanoparticles [10–12] have emerged as prime candidates due to their unusual optical properties and inherent biocompatibility.

Gold metal nanoparticles (NPs) exhibit narrow and intense absorption and scattering bands due to the phenomenon of plasmon resonance. This occurs at the resonance condition of the collective oscillation that the conduction electrons experience in an electromagnetic field of the appropriate wavelength [13]. The plasmon resonant condition of gold NPs depends upon their size, shape, structure (solid or hollow), and upon the refractive index of the embedding medium. Spherical gold nanoparticles have a single plasmon resonant extinction peak at around 520 nm, which does not shift extensively with changes in size and refractive-index of the surrounding medium. This is a wavelength at which light penetration in tissue is poor due to strong scattering and absorption by hemoglobin, and gold nanospheres are not useful in contrast enhancement for deep tissue imaging.

Rod-shaped NPs exhibit two plasmon resonances due to oscillation of the conduction electrons along the short axis as well as along the long axis of the particles. The former plasmon band is called the transverse resonance and the latter the longitudinal resonance. While the transverse plasmon band occurs in the neighborhood of 520 nm, the longitudinal band is red-shifted. The extent of the red-shift depends on the aspect ratio of the nanorod; the higher the aspect ratio, the further the shift. Thus by tailoring the length and/or width of these particles, their extinction peaks may be made to cover the low-energy visible to infrared wavelength regions.

The intense scattering and absorption of light, that occurs under the plasmon resonant condition coupled with the ability to tune the resonance into the near-infrared (NIR) by manipulating the aspect ratio, make gold nanorods extremely attractive as contrast agents for optical imaging techniques. Further, gold-protein chemistry is well developed and several bioconjugation protocols are available in the literature, which allows the combination of the targeting functionality of antibodies with such gold NPs. The inertness and biocompatibility of gold in general hold promise the use of gold NPs for in vivo imaging applications.

Gold NPs can be synthesized using wet chemical methods which are based on the reduction of gold salts by reagents such as sodium borohydride and ascorbic acid. Seed-mediated methods dominate wet chemical synthesis routes. These involve the reduction of gold using weak reducing agents onto small nanospheres of gold as seed, in the presence of shape-directing surfactants usually cetyl trimethylammonium bromide (CTAB). These methods may be distinguished into those that use silver ion assistance in growth solutions and those that do not.

Murphy and coworkers described the three-step growth protocol [14, 15], where medium to high aspect ratio nanorods could be synthesized, without the use of silver nitrate. Seed particles are generated by reducing gold salt using sodium borohydride in the presence of sodium citrate. The spheres are coated with a layer of negatively charged citrate ions that maintain colloid stability against aggregation by electrostatic repulsion. These spheres seed a growth solution comprising gold salt, CTAB, and ascorbic acid in three steps thereby slowing down reduction. The mechanism of nanorod formation by this method is not yet fully understood. Murphy et al. [15] proposed that the polar CTA^+^ head group of the surfactant binds with greater preference to certain crystallographic faces thereby passivating them to the deposition of gold. The other faces, on the other hand, would be exposed for gold to be reduced on, thereby producing anisotropic growth into rods.

The methods using silver nitrate in the growth solutions were proposed by Jana et al. [16], but modified by Nikoobakht and El-Sayed [17] to achieve spectacular yields of nanorods with excellent monodispersity. Importantly, they also showed that changing the quantity of Ag^+^ ions in the growth solution allows for fine-tuning of the aspect ratios of the nanorods. The mechanism at work in this protocol has been debated in the recent past. One mechanism postulates CTAB as a soft template which elongates on addition of Ag^+^ ions which occupy regions between the CTA^+^ head groups to reduce the repulsion between the head groups [17]. A second mechanism invokes the CTAB passivation concept with additional adsorption of silver bromide on facets slowing down reduction and producing rods shorter than those made without using Ag^+^ [18]. A third mechanism which has appeared recently [19] proposes the underpotential deposition of Ag^0^ on certain faces, followed by CTAB binding, which serves to stabilize the faces, and allows gold reduction on other faces resulting in rod formation.

In this article, we present our experiences in synthesizing gold nanospheres and nanorods using slight modifications of the protocols discussed above. Our goal is to obtain nanorods whose aspect ratios can be tuned to obtain plasmon peaks between 650 nm–850 nm. Next, we conjugate the gold nanospheres and gold nanorods to the HER81 monoclonal antibody using electrostatic and hydrophobic interactions. The conjugation does not use modifications of the bilayer charge of nanorods nor does it use any linkers. We discuss various aspects of these protocols and postulate a possible mechanism for the bioconjugation of the antibody with the gold nanorods. We also discuss the feasibility of using these molecular probes for contrast enhancement of photoacoustic cancer imaging using simulations.

## 2. EXPERIMENTAL SECTION: MATERIALS AND METHODS

### 2.1. Gold nanorods using the silver-assisted single surfactant growth method

As mentioned earlier, the seed-mediated protocol requires the use of small gold nanospheres to seed growth solutions with silver nitrate as per the protocol of Nikoobakht and El-Sayed [17].

The following are the reagents used for the synthesis of the gold seed and gold nanorods.

Tetrachloroauric acid (HAuCl_4_ ⋅ 3H_2_O) was purchased from Acros Organics (Belgium), hexadecyltrimethylammonium bromide (CTAB > 99%), sodium borohydride (NaBH_4_, 99%), and ascorbic acid (99%) from Aldrich (The Netherlands) and silver nitrate (AgNO_3_, 99.8%) from Merck (Germany). Prior to use, all glassware was cleaned with hydrofluoric acid (HF), further with aqua regia (HCl/HNO_3_) and rinsed twice with deionized water.

**Gold seed of 3.5 nm diameter**

The synthesis was done using protocols of Nikoobakht and El-Sayed [17] with slight modifications. A solution of CTAB (5 mL; 0.2 M) was sonicated for 20 minutes at 40°C in a water bath. A solution of HAuCl_4_⋅ 3H_2_O (5 mL; 0.0005 M) was added with continuous stirring under inert conditions (nitrogen environment). Then, an ice-cold aqueous solution of NaBH_4_ (0.6 mL; 0.01 M) was added at once with vigorous stirring for 1 minute. This seed solution (CTAB-capped) is further used during growth stage of nanorods.

**Gold nanorods of varying aspect ratios**

Five identical conical flasks containing 10 mL of a growth solution that consists of CTAB (5 mL; 0.2 M) and HAuCl_4_ ⋅ 3H_2_O (5 mL; 0.001 M) were prepared. The color of the growth solution is dark-yellow. AgNO_3_ (50, 100, 150, 200, and 250 *μ*L of 0.006 M) was added to the five identical growth solution flasks in an amount that was chosen so as to yield desired aspect ratios for the resulting nanorods. Following this, the mild reducing agent ascorbic acid (70 *μ*L; 0.1 M) was added to each growth solution conical flask to give colorless solutions. Finally, 14 *μ*L of preformed CTAB-capped seed solution was added to each conical flask, and mixtures were gently mixed. After 3 hours at 24°C, the nanorod suspension turned into a dark-blue solution with a brownish opalescence. These solutions were concentrated by centrifuging twice at 12000 g for 20 minutes which also enabled removal of the excess unbound CTAB. The centrifuged gold nanorods which are dispersed in water were stored at 4°c

### 2.2. Characterization of gold nanoparticles

Electron microscopy of the NPs was performed using a CM 30 Philips transmission electron microscope (TEM) or a Zeiss-1550 high-resolution scanning electron microscope (HRSEM). Particle sizes were estimated using NI Vision module (Labview, National Instruments) on the digital SEM images with at least 250 particles considered in each case. Extinction spectra of NPs (and bioconjugated NPs) were measured using the Shimadzu PC3101 UV-Vis-NIR spectrophotometer.

The concentration of nanorods synthesized was estimated using the relation *A* = *cdε*, where *A* is the measured absorbance, *c* the concentration in moles (M), *ε* the molar extinction coefficient (M^−1^ cm^−1^), and *d* the path length of the cuvette used to record the spectra. The derived molar extinction coefficients can be compared with *ε* values from a recent report, estimated for a range of aspect ratios of nanorods by measuring the gold content in sols using inductively coupled plasma (ICP) atomic emission spectroscopy [19].

### 2.3. Bioconjugation of HER81 mAb to gold nanoparticles

Conjugation was achieved using combination of electrostatic and hydrophobic binding interactions. The particles chosen for bioconjugation were 25 nm citrate-capped gold spheres (Aurion, Wageningen, The Netherlands), and silver-assisted surfactant mediated gold nanorods with aspect ratios of approximately 2.85 (see Table 1) with the longitudinal plasmon peak at 764 nm. The anti-HER2 mouse monoclonal antibody (mAb) (Immunicon, USA) was chosen as the targeting moiety. The antibody designated as HER81 recognizes Human EGF receptor 2, HER2. HER2, also called erbB2, is a member of the epidermal growth factor receptor (EGFR) family and is overexpressed in 20–40% of human breast cancers [20].

In general, for optimum conjugation, it is recommended that the pH of the antibody and gold sol be maintained at or slightly higher than the isoelectric point (pI) of the antibody [21]. The isoelectric (pI) point of HER81 mAb was determined using the Pharmacia PhastSystem isoelectric focusing (IEF). The pH of the antibody was adjusted with dialysis in 5mM sodium acetate buffer and the pH of the colloidal gold was adjusted with 0.1 M KOH, to approximately 0.5 pH units above this value.

Next, the minimum protecting amount of antibody to be used for the conjugation is determined. This is the amount of protein that is required to maintain colloidal stability of the conjugated NPs upon addition of NaCl [21] as judged by colorimetric analysis; as long as the conjugated NPs turns blue, particle aggregation takes place implying that the amount of protein is not sufficient to stabilize the suspension. By trial, different amounts of antibody are added to samples of the gold sol, gently mixed, and allowed to stand at room temperature for 2 minutes. Spectroscopic analysis reveals which sample remains stable; the minimum amount of protein added is then ascertained and is used for subsequent conjugation of the gold sol.

To block the free surfaces on the gold, 10% bovine serum albumin (BSA) in dialyzed buffer (sodium acetate), maintained at the same pH as the antibody solution, is used. The BSA was added to the conjugate to a final concentration of 1%and was allowed to incubate for 5 minutes. The resultant was then centrifuged for 30 minutes at 12000g to remove excess protein and incompletely stabilized particles. The resulting pellet is dispersed in phosphate buffered saline (PBS) containing 1% BSA and stored at 4°C.

### 2.4. Cell culture and cell-bioconjugate incubation

The HER2 positive mammary adenocarcinoma (SKBR3) cell line was used as a positive cell line; Chinese hamster ovary (CHO) cells were used as an HER2 negative cell line. The cells were cultured in RPMI 1640 medium (Invitrogen) supplemented with glutamine, 10% FBS (Fetal Bovine Serum) with antibiotics. Cells were maintained in an incubator at 37°C and 5% CO_2_.

The cells were detached from the tissue culture plate using trypsin. The cells were replated onto 12 mm glass cover slips in a 6-well tissue culture plate, and allowed to grow for 2 days at 37°C, 5% CO_2_. When the cells grew to 80% confluence on the cover slips, the cells were rinsed with phosphate buffered saline (PBS) and fixed in 4% paraformaldehyde (PFA) for 15 minutes at room temperature.

### 2.5 Immunostaining and confocal microscopy

After fixation, immunostaining was performed on the cells. The cells were incubated with 100 *μ*L of conjugated gold nanoparticles at a concentration of 9.7 × 10^10^ particles per mL for 2 hours followed by silver enhancement performed using a silver-staining kit (Aurion, Wageningen, The Netherlands).

Confocal microscopy reflection images of the cells on cover slips were recorded on a Zeiss LSM 510 confocal laser scanning microscope using a C-Apochromat 63 X/1.4 numerical aperture (NA) water-immersion objective. An excitation wavelength of 543 nm was chosen and reflection images recorded using a 500–550 nm bandpass filter. All images were acquired with pinhole diameters of 178 *μ*m. Care was taken to ensure that the excitation intensity as well as detector and amplifier gains were maintained at the same values for all images to facilitate comparison.

## 3. RESULTS

### 3.1 Synthesis of gold nanorods

**Figure 1:**
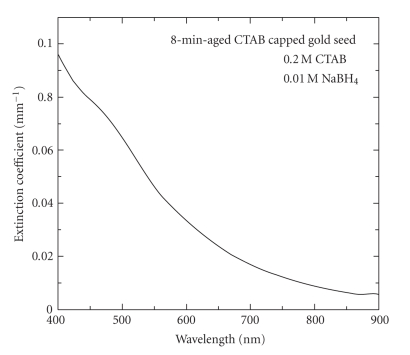
Optical extinction spectrum of preformed 8-minute-aged CTAB-capped gold nanospheres as seed for nanorod synthesis.

We used seed particles within about 8 minutes of formation in the subsequent growth phase. The optical extinction spectrum of 8-minute-aged seed is shown in Figure 1.

**Figure 2:**
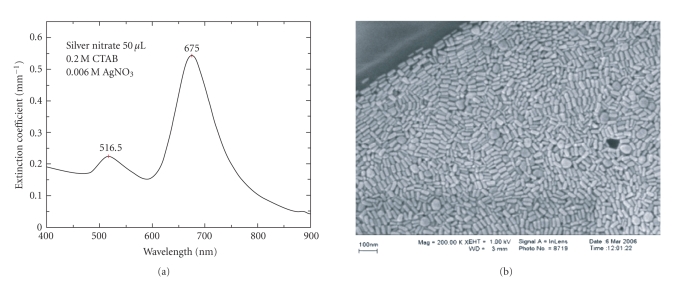
Gold nanorods synthesized using 50 *μ*L of silver nitrate in growth solution. (a) Optical extinction spectrum showing the transverse plasmon peak at 516.5 nm and the longitudinal plasmon peak at 675 nm. The amplitude of the longitudinal plasmon peak is higher than transverse plasmon peak which indicates the formation of high yield of nanorods compared to spheres. (b) High-resolution scanning electron microscope (SEM) image of gold nanorods showing high monodispersity. Few nanospheres are observed.

Figure 2 shows the extinction spectrum and HR-SEM image of the nanorods synthesized using 50 *μ*L AgNO_3_ in the growth solution. The peak at 675 nm can be attributed to longitudinal plasmon resonance and the peak in the vicinity of 516.5 nm to transverse plasmon resonance.

**Figure 3:**
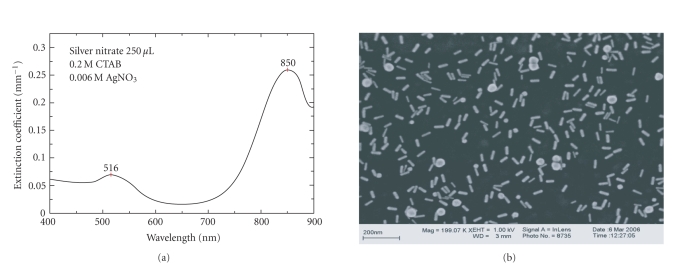
Gold nanorods synthesized using 250 *μ*L of silver nitrate in growth solution. (a) Optical extinction spectrum showing the transverse plasmon peak at 516 nm and the longitudinal plasmon peak at 850 nm. (b) High-resolution scanning electron microscope (SEM) image.

**Figure 4:**
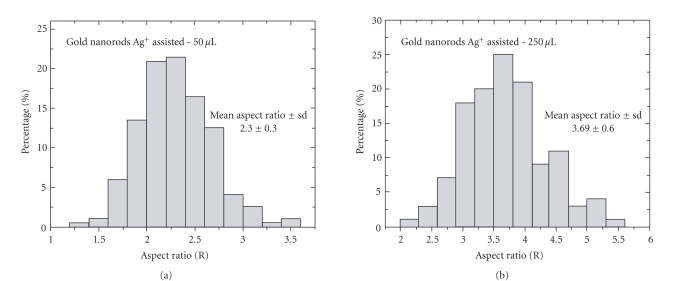
Histograms of gold nanorod aspect ratios synthesized with (a) 50 *μ*L silver nitrate, mean aspect ratio of 2.3 ± 0.3 (mean length 44.8 ± 4.1nm, mean width 19.8 ± 2.9 nm); and with (b) 250 *μ*L silver nitrate, mean aspect ratio of 3.6 ± 0.6 (mean length 51.0± 4.4 nm, mean width 14.1±2.1).

Examination of the SEM image and determination of the mean sizes confirm that the NPs produced consist of monodisperse nanorods of aspect ratio of 2.3 ± 0.3, with a small number of large spheres; the latter's extinction peak coinciding with the transverse plasmon band of the nanorods. Figure 3 shows the extinction spectrum and the SEM image for the sample produced using 250 *μ*L of silver nitrate. It is seen that the longitudinal plasmon band is shifted to 850 nm. Sizing from the SEM image yields an average aspect ratio of 3.6 ± 0.6. Figure 4 shows the size distribution of the 2 specimens.

The values of the molar extinction coefficient for the 2 cases above are 3.3±0.3×10^9^ and 5.5±0.3×10^9^ M^−1^ cm^−1^ obtained by extrapolation of the data as reported in [19]. With this, we arrive at the concentration of the nanorods with peak at 675 nm as 4.3±0.3×10^11^ NR/mL; for nanorods with the peak at 850 nm as 1.3±0.7×10^11^ NR/mL.

**Figure 5:**
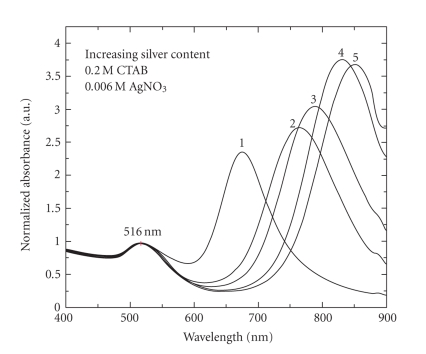
Normalized extinction spectra of gold nanorods with increasingly red-shifted longitudinal plasmon bands, synthesized using 50, 100, 150, 200, and 250 *μ*L of silver nitrate in the growth solution for curves 1–5, respectively. Normalization of the spectra is done with respect to the transverse plasmon peak amplitudes.

Figure 5 shows the consolidated normalized extinction spectra of 5 nanorod solutions, having identical growth solutions with varying silver nitrate volumes. The spectra were normalized to the peak at 516 nm, which is due to a combination of the transverse plasmon resonance of the nanorods and the signature peak of gold nanospheres. It is seen that with higher silver nitrate volumes the extent of red-shifting increases [22]. The details of the observed changes in aspect ratios and plasmon bands are presented in Table 1.

### 3.2 Bioconjugation of gold nanospheres and gold nanorods

A signature for successful binding of protein to gold NPs is a red-shifted and amplitude reduced plasmon band. Both these effects are due to the formation of the inhomogeneous layer of protein on the gold particle surface that leads to the modification of refractive index of the embedding medium.

**Figure 6:**
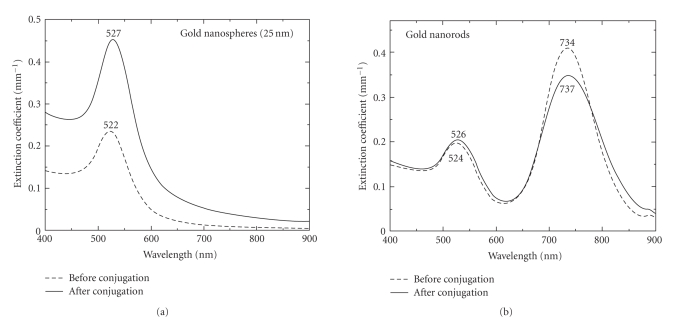
Extinction spectra before and after incubation of HER81 with (a) gold nanospheres, (b) gold nanorods. In both cases, a red shift in plasmon band(s) occurs after incubation with the antibody signifying successful bioconjugation.

Figure 6(a) shows the extinction spectrum of gold nanospheres before and after incubation with HER81. Figure 6(b) is the corresponding situation with gold nanorods before and after incubation with the HER81 mAb. In both cases, the characteristic red-shift in the extinction peak of the plasmon bands is seen.

Not too much should be read into the amplitude changes of the extinction spectra since centrifugation of the bioconjugate to remove unbound protein, redispersion in water, and other procedures results in a change in the concentration of the NPs used for spectroscopy.

**Figure 7:**
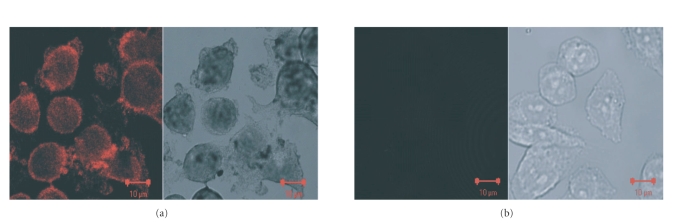
Confocal reflectance images (left) and bright field images (right) of (a) SKBR3 cells incubated with silver-stained HER81/gold sphere conjugates, (b) CHO cells under the same conditions. Care was taken to maintain the same acquisition parameters in both cases. The silver-stained bioconjugates are detected at the cell membranes of SKBR3 cells where HER2 is localized. This indicates successful conjugation and retention of functionality of the antibody after conjugation. No such accumulation of HER81/gold sphere conjugates is demonstrated in HER2 negative CHO cells.

Figure 7(a) show confocal reflectance image (on left) and bright field image (on right) of the HER81 mAb/gold sphere conjugates incubated with SKBR3 cells. As discussed in the experimental section, silver enhancement was used by which silver is reduced onto the gold particles forming large clusters around 500 nm in size. This then enables visualization under the microscope. The HER2 receptors are localized to the cell membranes of SKBR3 cells. The high intensities in both images at the cell membrane are then evidence of the preservation of the functionality of the antibody and also illustrate successful conjugation. The images in Figure 7(b), which show the situation with the negative control using the CHO cells, display no such accumulation of gold particles. Also, adding nonantibody conjugated gold NPs to the SKBR3 cells did not result in accumulation of the nanorods, indicating the specificity of the HER81 antibody-nanorod conjugate (data not shown).

**Figure 8:**
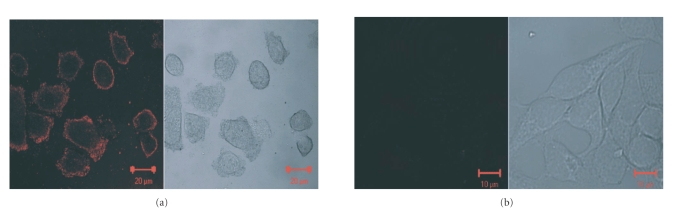
Corresponding images as in Figure 7 for bioconjugates consisting of silver-stained HER81/gold nanorod conjugates incubated with (a) SKBR3 cells and (b) CHO cells. Care was taken to maintain the same acquisition parameters in both cases. The bioconjugates are accumulated at the cell membranes of SKBR3 cells where HER2 is localized. As expected, no such accumulation takes place in the case of HER2 negative CHO cells.

Figures 8(a) and 8(b) are the results of the corresponding controls using the HER81 mAb/gold nanorods.

## 4. DISCUSSION

### 4.1 Gold nanorod synthesis

The end products of the seed-mediated growth protocols are crucially dependent on the nature of the seed, upon their size and upon the capping agents used. Additionally, the constituents and their concentrations in the growth solution influence the outcome of the synthesis products. The addition of silver ions in the growth solution and the use of preformed CTAB stabilized seed in the protocol of Nikoobakht and El-Sayed [17] produced not only a high yield of monodisperse nanorods but fine tunability of aspect ratios.

There are many unanswered questions regarding the mechanism of formation of gold nanorods using the silver-assisted protocol and this has been the topic of several studies [15, 17–19]. Recent reports of Orendorff and Murphy [19], and Liu and Guyot-Sionnest [23] provide some insights into the mechanisms that could be involved in the synthesis. It is postulated that silver ions are reduced by ascorbic acid even though it is a weak reducing agent, by the phenomenon of underpotential deposition (UPD). This is reduction of silver in monolayers on the growing gold nanorod surface at a potential less than the standard reduction potential [19]. The deposition is not uniform on the gold surface but occurs faster on the sidewalls compared with the end faces. Remarkably, the sidewalls in the case of nanorods produced with silver assistance using CTAB protected seed bear Au{110} faces, while the end faces have Au{100} faces. This is in contrast to the rods prepared by using citrate-capped seed without Ag^+^. This faster passivation of the sidewalls is followed by CTAB binding possibly via bromide ions. This inhibits the reduction of gold, which deposits on the end faces. Ultimately, the end faces are also stabilized preventing the formation of very long nanorods. The model claims also to explain the increase in aspect ratio of the nanorods produced with higher concentration of silver ions used, by proposing that higher UPD of silver monolayers occur on the sidewalls which one assumes reducing the width of the nanorods thus increasing the aspect ratios [19].

Indeed, we observe some phenomena that are consistent with the above model. We are able to synthesize gold nanorods only up to an aspect ratio of 3.6, as shown in Figure 3. Addition of higher volumes of silver nitrate produces no further increase in the aspect ratios of the particles. These particles have an average length of 51 nm. This supports the idea that ultimately complete passivation of the entire nanorod surface occurs preventing further gold deposition even though the reagents have not been exhausted. Further, we observe that nanorods that are made with increasing Ag^+^ volumes have smaller diameters with the lengths practically unchanged or only slightly increasing (see Table 1). The above model can also explain this. It must be mentioned that the model does not have an appealing explanation regarding the ability to tune the aspect ratios so precisely by Ag^+^ variation. It is very likely that the model will have to undergo refinements or even major changes before it is universally accepted.

### 4.2 Gold nanoparticle—antibody conjugation

The noncovalent conjugation of proteins to colloidal gold is usually due to a combination of electrostatic and hydrophobic interactions. Citrate-capped gold NPs are negatively charged due to a layer of negative citrate ions. Positively charged amino groups of the antibody will be attracted to the gold surface, and when the protein comes close enough for binding, the hydrophobic pockets of the protein will make contact and bind with the gold [24]. A general guideline to optimize the bioconjugation is that the pH of the antibody and the gold sol must be maintained at or slightly above the isoelectric point of the antibody [24]. Following this, procedure for citrate-capped gold nanospheres with the HER81 antibody resulted in good bioconjugation as shown by the spectroscopy and bioactivity studies (Figure 7).

With nanorods, the situation is more complex as compared with nanospheres. The sidewalls of the nanorods are expected to be stabilized with a bilayer of CTAB, which imparts a positive charge to the gold. Huang et al. [25] first changed the positively charged surface to a negatively charged one by exposing the nanorods to poly(styrenesulfonate) PSS polyelectrolyte solution. The PSS-capped nanorods were then treated in the same way as in Section 2.3 with the conjugation being done with anti-EGFR monoclonal antibodies.

Zeta potentials of the gold nanorod solution as originally prepared were determined to be +55 mV. Centrifugation to remove the excess unbound CTAB and redispersion of the rods in water saw a reduction in the zeta potential to +7.5 mV, which also points to a low stability. We surmise that inspite of the net positive charge, the unpassivated end faces would be negatively charged due to the presence of AuCl^−2^ ions [21]. We therefore performed the same protocol (as in Section 2.3) and found that the bioconjugation indeed was achieved as demonstrated by the red-shifted extinction spectra as seen in Figure 6. Further, confocal microscopy successfully detected the bioconjugates on the HER2 positive cell line (Figure 8), indicating the success of the conjugation.

We believe that the mechanism of conjugation is the same as that in the case of gold nanospheres that is electrostatic and hydrophobic physisorption. It is also likely that at the pH at which the antibody is maintained, the Fc fragment of the antibody that is rich in positively charged amino acids such as lysine will bind to the negatively charged chloride ion layer on the exposed end faces of the rods. We intend to perform studies that will elucidate this aspect. Further, we will perform the protocol of first capping the nanorods with PSS for example, and then comparing the antigen binding affinity constants of the bioconjugates from the two methods.

### 4.3 Potential contrast enhancing applications

The scattering and absorption bands of the synthesized nanorods span the wavelength regime between 675–850 nm that is of interest to optical imaging. This occupies the most important part of the “optical imaging window” where light penetration in tissue is high due to reduced scattering and absorption coefficients. Optical imaging techniques (Table 2) that rely on scattering and/or absorption contrast to detect pathological tissue could benefit from the use of such nanoparticles with or without targeting capability.

Our goal is to employ these particles as contrast agents for photoacoustic cancer imaging, which has been proposed earlier by Oraevsky and coworkers [26, 27]. Photoacoustic imaging relies on optical absorption for its signals. When photons are absorbed, nonradiative de-excitation of the absorbed optical energy takes place with the release of localized heat. The local thermal expansion that results produces pressure transients [5]. When illuminated with pulsed laser light, a tumor site by virtue of its higher absorption with respect to the healthy background tissue, due to angiogenesis [28], will act as a source of bipolar photoacoustic pulses. This ultrasound propagates with minimal distortion to the surface where it is detected using appropriate wideband detectors. The time-of-flight, amplitude, and peak-peak time of the bipolar PA pulse possess information regarding the location, absorption, and dimensions of the source, thereby permitting a reconstruction of the tumor site [29, 30].

It is known that the NIR optical absorption contrast of tumors versus healthy tissue, measured using optical mammographic methods, is between 1.5 and 3. Clinical trials of optical mammography are being conducted worldwide but at present, it seems implausible that intrinsic contrast alone will provide sufficient sensitivity and specificity, and targeted contrast enhancement is likely to be required [31]. Since the same contrast mechanism of optical absorption is operative in photoacoustic imaging as well, a similar conclusion may be anticipated.

An impression of the feasibility of using the nanorods synthesized for contrast enhancement is now discussed. The absorption cross-section of a nanorod at a wavelength, say 800 nm, is estimated using discrete dipole approximation (DDSCAT) simulations [32, 33] as *C*_abs_ = 2.8 × 10^−14^ m^2^ . A typical average optical absorption coefficient for an invasive ductal carcinoma is *μ*_a_ = 0.008 mm^−1^ at 800 nm. In order to achieve contrast enhancement, a certain number density of gold nanorods is required to exhibit higher absorption than the intrinsic value and may be calculated as

(1) $$\rho_{\text{NR}}\geq \frac{\mu_{a}}{C_{\text{abs}}}.$$

This gives *ρ*_NR_ = 2.8 × 10^8^ NR/cm^3^ . Further photoacoustic signals can be enhanced by a thermal nonlinearity mechanism to 3 orders of magnitude higher [34], then the modified number density of nanorods required is only *ρ*_NR_ = 2.8 × 10^5^ NR/cm^3^ .

Published studies report that most tumour cell types express from 2 × 10^4^ to 20 × 10^4^ ErbB2 receptors/cell [12]. Let us assume arbitrarily that 2 × 10^3^ of these sites per cell are occupied by conjugated nanorods. Further, if we assume that 1% of cells at a tumor site overexpress HER2 results in a figure of 2 × 10^6^ cancer cells/cm^3^ . This will then lead to an estimation of the density of binding sites of the order of 10^9^ cm^−3^ . Comparison of *ρ*_NR_ and the estimated figure of density of binding sites leads us to believe that contrast enhancement will be possible.

We intend to test these molecular probes in small animal photoacoustic imaging. Contrast enhancement with untargeted PEG-coated nanoparticles will be studied. Accumulation of the contrast agent at the tumor site will depend on enhanced permeation and retention (EPR). Active targeted studies will follow, with conjugated nanoparticles administered to the animal via the tail vein. In all studies, emphasis will be on ascertaining the sensitivity/efficacy of the technique with and without contrast agent.

## 5. CONCLUSIONS

We have synthesized gold nanorods with optical extinction peaks in the region from 675–850 nm making these eminently suited for scattering and absorption contrast enhancements in optical imaging. We have performed bioconjugation of these nanorods with HER81 antibodies, which bind with high efficiency to HER2 receptors expressed by SKBR3 breast carcinoma cells. We demonstrated in fixed cell studies that the targeting functionality of the antibody moiety remains viable. However, it must be mentioned that the situation *in vivo* will be complex compared to the simplified situation *in vitro* . Other unresolved issues remain at present. One of these is regarding toxicity and cellular uptake of these particles *in vivo* . Further, whether these molecular probes will be able to extravasate into the tumor tissue through leaks in the vasculature has not yet been studied. These are some lines of research that we intend to follow in the near future.

## Figures and Tables

**Table 1:** Mean aspect ratios, lengths, widths, and longitudinal plasmon peaks for nanorods synthesized using the silver-assisted growth method.

Sample	Volume of AgNO_3_ ( *μ*L)	Aspect ratio (R)	Length (nm)	Width (nm)	LP band (nm)
1	50	2.3±0.3	44.8±4.1	19.8±2.9	675
2	100	2.85±0.6	45.1±5.5	15.8±3.1	764
3	150	3.0±0.6	41.7±3.9	13.9±2.3	788
4	200	3.1±0.5	52.0±4.6	16.8±2.8	831
5	250	3.6±0.6	51.0±4.4	14.1±2.1	850

**Table 2:** Important optical imaging techniques that utilize absorption and scattering contrasts in biology and medicine.

Technique	Imaging depth	Imaging resolution	Mechanism	Typical imaging applications
Confocal microscopy [1]	500 *μ*m	> 250 nm	Scattering / absorption	Tissue surfaces
Two-photon microscopy [2]	800 *μ*m	> 250 nm	Absorption	Tissue surfaces
Optical coherence tomography [3]	2 mm	1 *μ*m	Scattering	Surfaces/subsurfaces of tissue
Diffuse optical tomography [4]	> 20 mm	*≈* 10 % depth	Scattering/absorption	Small animal;human breast and brain
Photoacoustic imaging [5]	> 20 mm	*<* 1 mm(detector bandwidth limited)	Absorption	Subsurface to deep imaging;small animal; human breast
